# Diversification of an emerging bacterial plant pathogen; insights into the global spread of *Xanthomonas euvesicatoria* pv. *perforans*

**DOI:** 10.1371/journal.ppat.1013036

**Published:** 2025-04-09

**Authors:** Sujan Timilsina, Fernanda Iruegas-Bocardo, Mustafa O. Jibrin, Anuj Sharma, Aastha Subedi, Amandeep Kaur, Gerald V. Minsavage, Jose C. Huguet-Tapia, Jeannie Klein-Gordon, Pragya Adhikari, Tika B. Adhikari, Gabriella Cirvilleri, Laura Belen Tapia de la Barrera, Eduardo Bernal, Tom C. Creswell, Tien Thi Kieu Doan, Teresa A. Coutinho, Daniel S. Egel, Rubén Félix-Gastélum, David M. Francis, Misrak Kebede, Melanie Lewis Ivey, Frank J. Louws, Laixin Luo, Elizabeth T. Maynard, Sally A. Miller, Nga Thi Thu Nguyen, Ebrahim Osdaghi, Alice M. Quezado-Duval, Rebecca Roach, Francesca Rotondo, Gail E. Ruhl, Vou M. Shutt, Petcharat Thummabenjapone, Cheryl Trueman, Pamela D. Roberts, Jeffrey B. Jones, Gary E. Vallad, Erica M. Goss

**Affiliations:** 1 Department of Plant Pathology, University of Florida, Gainesville, Florida, United States of America; 2 Department of Crop Protection, Ahmadu Bello University, Zaria, Nigeria; 3 Southwest Florida Research and Education Center, University of Florida, Immokalee, Florida, United States of America; 4 Department of Horticultural Science, North Carolina State University, Raleigh, North Carolina, United States of America; 5 Department of Entomology and Plant Pathology, North Carolina State University, Raleigh, North Carolina, United States of America; 6 Dipartimento di Agricoltura, Alimentazione e Ambiente, Sezione Patologia Vegetale, Catania, Italy; 7 Centro de Investigacíon an Alimentación y Desarrollo, Culiacán, Sinaloa, Mexico,; 8 Department of Horticulture and Crop Science, The Ohio State University, Wooster, Ohio, United States of America; 9 Botany and Plant Pathology Department, Purdue University, West Lafayette, Indiana, United States of America; 10 Department of Plant Protection, College of Agriculture, Can Tho University, Can Tho, Vietnam; 11 Department Biochemistry, Genetics and Microbiology, Centre for Microbial Ecology and Genomics, Forestry and Agricultural Biotechnology Institute, University of Pretoria, Pretoria, South Africa; 12 Departamento de Ciencias Naturales y Exactas, Universidad Autónoma de Occidente, Unidad Regional Los Mochis, Los Mochis, Sinaloa, México; 13 Biotechnology Department, Collage of Biological and Chemical Engineering, Addis Ababa Science and Technology University, Addis Ababa, Ethiopia; 14 Department of Plant Pathology, The Ohio State University, Wooster, Ohio, United States of America; 15 Department of Plant Pathology, China Agricultural University, Beijing, China; 16 Department of Horticulture and Landscape Architecture, Purdue University, West Lafayette, Indiana, United States of America; 17 Department of Plant Protection, College of Agriculture, University of Tehran, Karaj, Iran; 18 Laboratorio de Fitopatologia, Embrapa Hortalicas, Brasilia-DF, Brazil; 19 Queensland Department of Agriculture and Fisheries, Brisbane, Queensland, Australia; 20 Department of Plant Agriculture, Ridgetown Campus, University of Guelph, Ridgetown, Ontario, Canada; 21 Department of Plant Science and Biotechnology, University of Jos, Jos, Nigeria; 22 Gulf Coast Research and Education Center, University of Florida, Wimauma, Florida, United States of America; 23 Division of Entomology and Plant Pathology, Faculty of Agriculture, Khon Kaen University, Khon Kaen, Thailand; 24 Emerging Pathogen Institute, University of Florida, Gainesville, Florida, United States of America; CAU: Christian-Albrechts-Universitat zu Kiel, GERMANY

## Abstract

Emerging and re-emerging plant diseases continue to present multifarious threats to global food security. Considerable recent efforts are therefore being channeled towards understanding the nature of pathogen emergence, their spread and evolution. *Xanthomonas euvesicatoria* pv. *perforans (Xep*)*,* one of the causal agents of bacterial spot of tomato, rapidly emerged and displaced other bacterial spot xanthomonads in many tomato production regions around the world. In less than three decades, it has become a dominant xanthomonad pathogen in tomato production systems across the world and presents a compelling example for understanding diversification of recently emerged bacterial plant pathogens. Although *Xep* has been continuously monitored in Florida since its discovery, the global population structure and evolution at the genome-scale is yet to be fully explored. The objectives of this work were to determine genetic diversity globally to ascertain if different tomato production regions contain genetically distinct *Xep* populations, to examine genetic relatedness of strains collected in tomato seed production areas in East Asia and other production regions, and to evaluate variation in type III secretion effectors, which are critical pathogenicity and virulence factors, in relationship to population structure. We used genome data from 270 strains from 13 countries for phylogenetic analysis and characterization of type III effector gene diversity among strains. Our results showed notable genetic diversity in the pathogen. We found genetically similar strains in distant tomato production regions, including seed production regions, and diversification over the past 100 years, which is consistent with intercontinental dissemination of the pathogen in hybrid tomato production chains. Evolution of the *Xep* pangenome, including the acquisition and loss of type III secreted effectors, is apparent within and among phylogenetic lineages. The apparent long-distance movement of the pathogen, together with variants that may not yet be widely distributed, poses risks of emergence of new variants in tomato production.

## Author summary

Bacterial diseases pose significant threats to food security by reducing crop yield and increasing production costs. Managing these diseases is particularly challenging when pathogen populations are genetically diverse, rapidly evolving, and capable of long-distance dispersal. *Xanthomonas euvesicatoria* pv. *perforans* (*Xep*), one of the pathogens responsible for bacterial spot disease of tomato, exemplifies these challenges. Since its discovery in Florida in 1991, *Xep* has spread globally, affecting tomatoes in warm and wet production regions worldwide. Previous studies have indicated genetic diversity within *Xep*, suggesting the emergence of multiple lineages, but there was not a comprehensive global analysis. Here, we examined genome sequences of *Xep* strains from five continents and discovered extensive genetic diversity, including in genes important for virulence and breeding for resistance in tomatoes. In addition, we found that genetically similar strains were present on different continents, likely due to the international movement of contaminated seeds through global production chains. Our findings underscore the need for periodic monitoring of these pathogen populations and new approaches to effectively manage bacterial spot disease of tomato.

## Introduction/Main

Emerging and re-emerging plant diseases are a constant threat to global food security [1–3]. Bacterial plant pathogens cause some of the most intractable diseases of crops worldwide [4–7]. Novel emergence and re-emergence of bacterial diseases continue to be reported across the globe and are associated with an upsurge in efforts devoted to understanding the nature of pathogen emergence, spread, and evolution [8–17]. A bacterial plant pathogen that emerged in the last few decades and is of global epidemiological consequences is *Xanthomonas euvesicatoria* pv. *perforans* (*Xep*) [18] (syn. *X. perforans* [19, 20])*,* one of the causal agents of bacterial spot of tomato [21].

Bacterial spot disease of tomato affects all aboveground plant parts including leaves, stems, flowers and fruit. Under optimal environmental conditions, fruit lesions and/or extensive defoliation can dramatically limit marketable yields and pose a continuous challenge to tomato production [22–24]. Once epidemics are initiated, growers have limited management tools and have relied heavily on copper-based bactericides. However, reliance on copper compounds has led to widespread copper tolerance [25–33]. Alternative bactericides are often costly, provide insufficient control when the weather favors rapid disease development, and rarely improve yields. While historically four taxa have caused this disease, *Xep* has emerged rapidly and become a major player in tomato [21,30,34–41]. *Xep* was first reported in 1991 in Florida, USA [19] and is now found in all tomato production areas of the world, including regions with no history of the disease [42]. *Xep* has been isolated from tomato seed [19]; therefore, a likely hypothesis for new outbreaks of *Xep* is pathogen movement with seeds and planting materials [43].

Tomato production is characterized by a high seed replacement rate (99.3%), meaning that growers require seeds each season, which in turn requires large-scale seed production [44]. Tomato hybrid seed production is concentrated in geographic areas where environmental conditions minimize seed contamination by pathogens and seed production costs are low. These seed production regions supply hybrid seeds globally for commercial production of tomato fruits for the fresh market or for processing into tomato products (e.g., sauce, paste, and diced tomatoes). The long-distance movement of seeds poses a high risk for dissemination of seed borne pathogens to commercial tomato production areas. Seedlings are typically grown in transplant facilities and then transplanted into fields for the regional and international transplant markets, potentially amplifying and further disseminating seed-borne pathogens [45].

The success of *Xep* as a pathogen has been attributed to its production of bacteriocins against competing bacterial spot species, rapid genome evolution via recombination, and introduction of genes via horizontal gene transfer that contribute to fitness in tomato fields [46–52]. Distinct genetic lineages of *Xep*, each with unique patterns of allelic variation among core genes (genes present in all strains), were identified in fresh market and processing tomato production fields in the United States [47,50,51,53,54]. Additional lineages of *Xep* were found in Nigeria, Iran, Italy, and the Southwest Indian Ocean islands [42,48,55].

*Xep* strains, like other xanthomonads, acquire nutrients through colonization of susceptible hosts. The type III secretion system (T3SS) and type III effector (T3E) proteins are critical for suppression of host defenses and virulence by *Xep* [56]. Effector content varies among *Xanthomonas* species and distinct lineages of *Xep* have distinguishable effector content [26,48,50,57,58]. Strains of *Xep* isolated in the 1990s were unable to cause disease on pepper [59], but now strains of *Xep* are causing bacterial spot disease of pepper [50,58,60]. Host range expansion was attributed, in part, to loss of effectors that act as avirulence factors in pepper and other genomic changes as a result of recombination with other *Xanthomonas* lineages. Effector variation can cause differences in disease epidemiology in addition to host range [49,57,61]. For example, wildtype strains with the acquired effector XopJ2 showed three times faster spread in the field than isogenic mutant strains without the effector [51].

Emerging pathogens can show limited genetic variation if they experienced a bottleneck during the ecological and evolutionary processes that often precede emergence (e.g., host jump or introduction event) [62]. *Xep* appears genetically diverse but it is not known how this variation is structured across global tomato production regions. The first objective of this work was to determine if different tomato production regions contain genetically distinct *Xep* populations. Second, we asked if there was evidence for long-distance pathogen dissemination, as would be indicated by genotypes shared among distant regions. Specifically, we obtained strains from tomato seed production areas in East Asia and asked if they resembled strains from fruit production fields elsewhere in the world, which would be expected if strains are being disseminated in seeds. Third, we estimated the timing of *Xep* population expansion relative to its first report in 1991. Finally, we evaluated T3E content and allelic variation in the context of geography and core genome variation. Overall, we found extensive genetic diversity within *Xep;* genetically similar strains in distant geographic regions, inclusive of seed production regions; evidence of diversification prior and subsequent to the first report of emergence; and lineage-specific T3E repertoires. Together, these results illustrate the capacity for this pathogen to rapidly evolve and strongly support the potential for intra- and intercontinental movement of pathogens in tomato production systems.

## Results

### X. *euvesicatoria* pv. *perforans* strains from seed and commercial fruit production areas

A total of 270 *Xep* genomes from 13 different countries – representing seed and fruit production – were used in this study (Table 1). We generated new genome sequence data for 153 strains of these strains and used published data for 117 (See accessions in S1 Table). *Xep* strains were differentiated from other tomato-pathogenic xanthomonads using a real-time qPCR assay that specifically amplifies the *hrcN* (*hrpB7*) gene in *Xep* [63] and inoculated on tomato cv. ‘Bonny Best’ to confirm pathogenicity. Strains from China, Thailand, and Vietnam were collected from seed production areas (n = 31) and all other strains (n = 239) were collected in commercial fruit production areas from Australia, Brazil, Canada, Ethiopia, Iran, Italy, Mexico, Nigeria, South Africa, and the United States. Within the US, strains were collected from seven different states in the Midwest and Southeast, including strains collected since 1991 from Florida.

**Table 1:** *Xanthomonas euvesicatoria* pv. *perforans* strains used in this study.

Country	Locality	Year	Strain (original name, if applicable)
Australia [57]	Queensland	2015	Aus3, Aus7, Aus14
		2016	Aus5, Aus10, Aus11
		2017	Aus1, Aus15, Aus16
Brazil [39]	São Paulo	2011	Bzl1 (2011-107), Bzl2 (2011-132)
	Goiás	2012	Bzl3 (2012-08)
	Goiás, São Paulo	2013	Bzl5 (2013-16), Bzl6 (2013-42)
	Goiás, Minas Gerais	2014	Bzl7 (2014-10), Bzl8 (2014-17)
	Minas Gerais	2015	Bzl10 (2015-53), Bzl11 (2015-56)
	Goiás	2016	Bzl13 (2016-08)
	Goiás	2017	Bzl14 (2017-21)
Canada	Ontario	2016	4A, 4D, 12A, 14A
China		2016	CHI-3, CHI-5, CHI-6, CHI-7, CHI-8, CHI-10, CHI-12, CHI-15, CHI-18
Ethiopia [36]		2011	ETH5, ETH11, ETH21, ETH25, ETH33
Iran [41]		2013	K41, F210, F215, TOM801, TOM816
Italy [64]		2011	1P6S1, 2P4S1, 2P4S1D, 2P6S1, 1P4S1D
Mexico			Mexico-1, Mexico-3, Mexico-LT1, Mexico-LT3, Mexico-LT5
Nigeria [37,65]		2014	NI-1, NI-2, NI-4, NI-7, NI-12, NI-13
		2015	KS3, KS5, KS9, KS28
South Africa	Pretoria		X2-B14, X10-B85, X59-BD1351, X47-BD167
Vietnam			SEA-3, SEA-5, SEA-21, SEA-23
Thailand		2016	THA-8, THA-14, THA-40, THA-45, THA-54, THA-72, THA-81A, THA-100, THA-112, THA-116, THA-119, THA-120, THA-126, THA-127, THA-128, THA-132, THA-135, THA-157A
United States	Alabama [66]	1996	Xp1861
	Indiana [28]	2016	16-1165A1, 16-1181-2, 16-1182A, 16-1184A, 16-1187A, 16-1205A, 16-1402A, 16-974C, 16-990A, 16-990C
		2014	14-463-1A
	Florida [19,20,43,47,58,67]	1991	XV0938, Xp91-118, Xp894, Xp909, Xp1183
		1992	Xp1118, Xp1144
		1993	Xp1241, Xp1268, Xp1275
		1994	Xp1550, Xp1564
		1995	Xp1797, Xp1805
		1996	Xp1856
		1997	Xp1912
		1998	Scott-1, Xp1920
		2006	Xp1-5, Xp1-6, Xp3-12, Xp3-15, Xp3-16, Xp3-8, Xp4-20, Xp5-14, Xp5-6, Xp5-9, Xp7-12, Xp8-16, Xp9-5, Xp10-13, Xp11-2, Xp15-11, Xp17-12, Xp18-15
		2007	Xp4B
		2010	Xp2010
		2011	GEV485
		2012	GEV839, GEV872, GEV893, GEV904, GEV909, GEV915, GEV917, GEV936, GEV940, GEV968, GEV993, GEV1001, GEV1026, GEV1044, GEV1054, GEV1063
		2013	TB6, TB9, TB15
		2015	GEV2047, GEV2048, GEV2049, GEV2050, GEV2052, GEV2055, GEV2058, GEV2059, GEV2060, GEV2063, GEV1989, GEV1991, GEV1992, GEV1993, GEV2004, GEV2009, GEV2010, GEV2011, GEV2013, GEV2015, GEV1911, GEV1912, GEV1913, GEV1914, GEV1915, GEV1916, GEV1917, GEV1918, GEV1919, GEV1920, GEV1921
		2016	GEV2065, GEV2067, GEV2072, GEV2087, GEV2088, GEV2089, GEV2097, GEV2098, GEV2099, GEV2108, GEV2109, GEV2110, GEV2111, GEV2112, GEV2113, GEV2114, GEV2115, GEV2116, GEV2117, GEV2118, GEV2119, GEV2120, GEV2121, GEV2122, GEV2123, GEV2124, GEV2125, GEV2126, GEV2127, GEV2128, GEV2129, GEV2130, GEV2132, GEV2133, GEV2134, GEV2135
	Louisiana [27]	2013	mli-2
	North Carolina [30]	2015	NC-14, NC-47, NC-67, NC-101, NC-112, NC-204
		2016	NC-242, NC-252, NC-282, NC-289, NC-350, NC-373, MRS-30P-011
	South Carolina [43]	2016	GEV2407, GEV2408, GEV2384, GEV2388, GEV2389, GEV2390, GEV2391, GEV2392, GEV2393, GEV2396, GEV2397, GEV2399, GEV2400, GEV2403, GEV2410, GEV2420
	Ohio [53]	2017	SM-1806, SM-1807, SM-1808, SM-1809, SM-1810, SM-1811, SM-1812, SM-1813, SM-1814, SM-1815, SM-1828, SM-1829, SM-1830, SM-1831

### Genomic diversity in *X. euvesicatoria* pv. *perforans*

To examine genetic diversity in the core genome, we curated a set of 887 genes that were present in all 270 *Xep* genomes based on IMG/JGI gene annotation. The aligned sequence length of concatenated core genes was 617,855 bp, which contained 14,427 polymorphic sites after removing ambiguous nucleotides and any alignment gaps (S1 Data). Grouping strains by state within the United States and country elsewhere produced an FST [68] of 0.66. The values of Watterson’s θ per site for the entire 617,854 bp alignment by geographic location, when represented by more than one strain, ranged from 9.16 × 10^-6^ (15 SNPs across the core gene alignment) to 0.00313 (5929 SNPs) (S2 Table). Nucleotide diversity (average number of differences per site, [69]) ranged from 5.50 × 10^-6^ to 0.00208. Both extremes in diversity came from the Midwestern U.S., Ohio and Indiana respectively (S2 Table). Tajima’s D [70] by geographic location ranged from –2.06 to 1.71, but many locations had low sample sizes (S2 Table).

**Fig 1:**
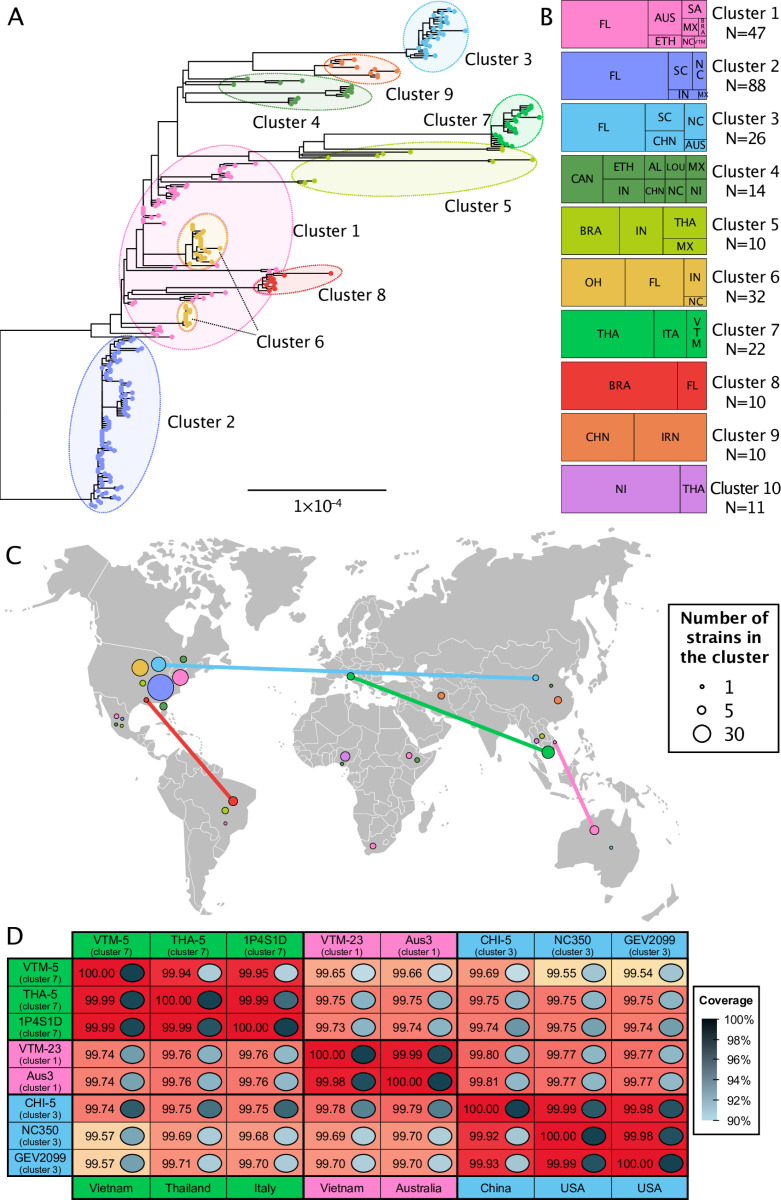
Population structure of *Xanthomonas euvesicatoria* pv. *perforans* strains collected from tomato production regions. (A) Maximum likelihood phylogenetic tree of 259 *X. euvesicatoria* pv. *perforans* strains constructed with nucleotide sequences from 887 core genes, corrected for recombination by ClonalFrameML. Tips are colored according to clusters identified by hierBAPS. Nucleotide alignment is available as S1 Data. (B) Distribution of 270 strains in each cluster by country or state of collection. Strains designated as cluster 10 (n=11) were genetically distant and excluded from the tree and hierBAPS analysis (see S1 Fig). N indicates total number of strains in each cluster. Geographic abbreviations are as follows: AUS – Australia; BRA – Brazil; CAN – Canada; CHN – China; ETH – Ethiopia; FL – Florida, USA; IN – Indiana, USA; IRN – Iran; ITA – Italy; LOU – Louisiana, USA; MX – Mexico; NC – North Carolina, USA; NI – Nigeria; AL – Alabama, USA; OH – Ohio, USA; SA – South Africa; SC – South Carolina, USA; THA – Thailand; VTM – Vietnam. (C) Map showing distribution of clusters by country of collection. Lines show instances of strains with high core gene sequence identity that were collected in different countries (S3 Table). Base layer of the map is courtesy of Eurostat (https://ec.europa.eu/eurostat/web/gisco). (D) Pairwise comparison of whole genome average nucleotide identity (ANI) confirmed high identity between strains isolated from different continents. For each comparison, genome coverage is shown by grayscale in boxes, scale shown to the right. Values for each comparison are for genomes in rows when compared to genomes in columns. See S3 Table for additional ANI output.

Maximum likelihood phylogenetic analysis of core SNPs revealed diversifying lineages of *Xep* (Fig 1) and an especially diverged lineage of 11 strains from Nigeria and Thailand (S1 Fig) that included a previously defined atypical strain – NI1 – from Nigeria [48]. After excluding the 11 atypical strains due to their phylogenetic divergence, ClonalFrameML [71] estimated an overall ratio of recombination rate to mutation rate (R/theta) of 0.60, with recombination causing approximately seven times more base changes than mutation (delta = 231; nu = 0.05). There were an estimated 221 recombination events that affected more than 96 Kbp in terminal branches and 494 recombination events detected in internal branches encompassing 190 Kbp.

To summarize population structure based on core gene SNPs, we used hierBAPS [72], which assigned individual strains to 9 clusters using allele frequencies (Fig 1 and S1 Table). This analysis did not include the 11 highly diverged strains from Nigeria and Thailand, which we designated as cluster 10. F_ST_ among clusters was 0.80. In some cases, clusters corresponded to phylogenetic lineages, including clusters 2, 3, 7, 8, and 9 (Fig 1). The remaining clusters were polyphyletic, encompassing multiple diverged clades or individual strains. Nucleotide diversity within clusters ranged from 2.23 × 10^-5^ for cluster 8 to 0.0011 for cluster 5, while cluster 3 had the highest diversity for a monophyletic cluster (6.98 × 10^-4^) (S2 Table). Watterson’s θ per site was lowest for cluster 8 (3.57 × 10^-5^) and highest for cluster 6 (0.0018), cluster 3 (0.0013), and cluster 5 (0.0011) (S2 Table). Analysis of presence-absence gene variation in the pangenome showed that polyphyletic clusters 1, 4, and 5 had the most variation in gene content (S2 Fig).

### Geographic distribution of *X. euvesicatoria* pv. *perforans* core gene clusters

Cluster 1 encompasses genetically diverse strains from seven countries, including most of the strains from Australia, all four strains from South Africa, and one strain from Southeast Asia (Fig 1B). All USA strains assigned to cluster 1 were isolated in or before 2006 from Florida except for one strain from North Carolina. Cluster 2 contains 88 strains from the United States and one from Mexico, while Cluster 3 includes strains isolated from Florida, North and South Carolina, China, and Australia. Cluster 4 encompasses multiple lineages of strains from the United States, Canada, Ethiopia, China, and Nigeria. Cluster 5 is polyphyletic with diverged strains from the United States, Mexico, Brazil, and Thailand. Cluster 6 was isolated only within the United States from Florida, Indiana, North Carolina, and Ohio. Cluster 7 is a monophyletic group of strains from Southeast Asia and Italy. Cluster 8 is another monophyletic group found only in Brazil and Florida. Cluster 9 includes two clades of strains, one from China and the other from Iran and Nigeria. Cluster 10 comprises the atypical strains from Nigeria and similar strains from Thailand. Most countries contained strains from more than one core gene cluster (Fig 1C).

Clusters 1, 3, 4, 5, 7, 9, and 10 contain strains isolated from both seed production and commercial fruit production regions, whereas strains in clusters 2, 6, and 8 were only isolated from commercial fruit production regions. Some strains found on different continents were nearly identical in core gene sequences with very high average nucleotide identity (Fig 1D). Strains in cluster 1 from Australia differed by 6 to 10 SNPs in more than 617 Kbp of core gene sequence from strain VTM-23 from Vietnam. Whole genome pairwise average nucleotide identity (ANIb) between VTM-23 and Aus3 was 99.99% compared to ANIb values ranging from 99.65 to 99.81 for comparisons to genomes representing other clusters (Fig 1D). Strains from the USA had up to 99.87 ANIb with strains from Australia and Vietnam (S3 Table). A different strain from Vietnam, VTM-5 in cluster 7, had as few as four SNPs in the core genome when compared to strains from Italy and ANIb of 99.95% to Italian strain 1P4S1D (Fig 1D). Likewise, strains collected in a seed production region in China had ANIb up to 99.99% with strains from Florida and North Carolina. We also found similar strains between Brazil and USA, for example Bzl-10 (Minas Gerais) and Xp3-8 (Florida) had greater than 99.9% ANI (S3 Table). Other strains were similar between countries in core genes only after correction for recombination.

### Timing of *X. euvesicatoria* pv. *perforans* lineage emergence

**Fig 2:**
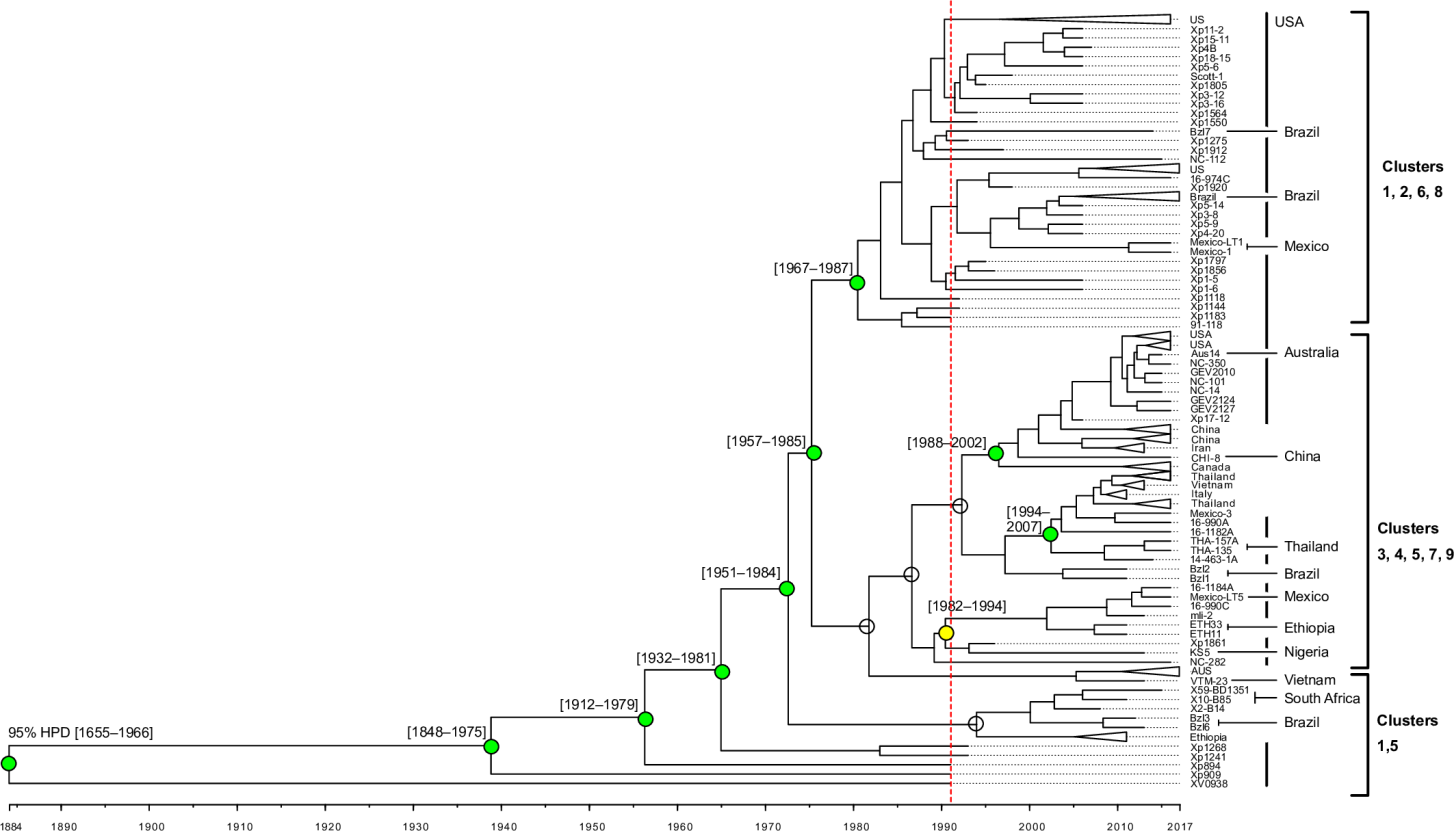
Dated phylogeny of 259 *X. euvesicatoria* pv. *perforans* strains. BactDating analysis estimated an approximately 130-year history for *Xep* strains in core gene clusters 1 through 9 (Fig 1). Red dotted line indicates the first documented isolations in 1991. Internal nodes were collapsed for clades containing strains from a single country with branch tips indicating country or strain (for full tree see S4 Fig). Bold vertical lines to the right of tip labels indicate strains from USA; other countries are labeled. Temporal signal was assessed using Phylostems and results are shown for major nodes (for full results see S3 Fig). Empty circles indicate no significant temporal signal. Colored circles indicate nodes with statistically significant temporal signal based on adjusted R^2^ values: green – 0.13-0.19; yellow – 0.45. The 95% highest posterior density (95% HPD) of date estimates for major nodes with significant temporal signals are shown in brackets.

We used the years of strain collection to estimate the timing of diversification of our sample of *Xep*, excluding the cluster 10 strains. We inferred dated phylogenies using whole genome alignments with inferred recombinant sites removed by Gubbins [73]. Due to recombination with other *X. euvesicatoria* lineages, we did not include an outgroup (S1B Fig; [48]). Sampling year was significantly correlated with root-to-tip distance (R^2^ = 0.20 for the whole genome alignment, *P* < 1×10^-4^, S3 Fig). The root inferred by the BactDating R package [74] was placed between strains isolated in Florida in 1991. The most recent common ancestor (MRCA) of all strains was dated to 1884 (95% HPD: 1655–1966). Notably, strains that were isolated in the early 1990s, when *Xep* was first detected in U.S. tomato production [19,37], represented multiple lineages (Fig 2). The MRCA of the clade representing core gene clusters 1, 2, 6, and 8 (including strains from USA, Brazil, and Mexico) was dated to 1980 (95% HPD: 1967–1987). A major clade, encompassing strains in clusters 3, 4, 5, 7, 9, which were collected in Africa, the Americas, Asia, Australia, and Europe, did not have a significant temporal signal across the clade. We repeated the analysis with BEAST, which inferred a different rooting. The tree inferred by BEAST placed the root between two strains isolated in 2011 from Brazil and all other strains (S4B Fig). The MCRA of the BEAST tree was dated to 1868 (95% HPD: 1862–1919), which was similar to the root date estimated using BactDating (S4 Fig).

### Type III effector content

**Fig 3:**
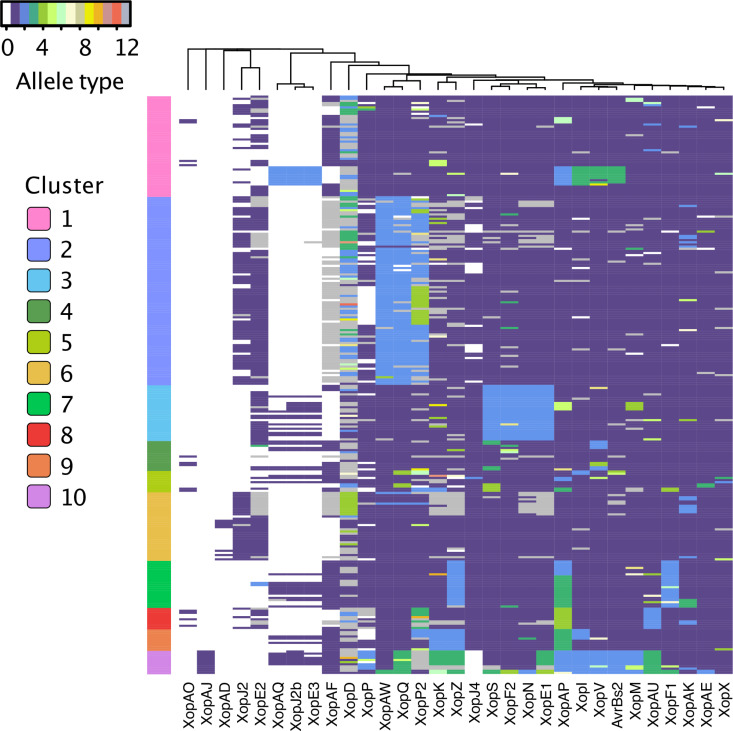
Variation in type III effectors (Xop proteins) in *Xanthomonas perforans.* Type III effectors are in columns and 270 *X. euvesicatoria* pv. *perforans* strain in rows. Effector status is shown by allele type: absence is indicated by allele type 0 (white), while the most frequent allele observed when the effector is present is allele type 1 (purple), second most frequent is allele type 2 (blue), and so on. Putative pseudogenized effectors are shown as allele 13 (gray). The order of columns was determined by hierarchical clustering analysis, placing similarly distributed effectors adjacent to each other. Genomes showing BLAST hits to TAL effector(s) are indicated in S3 Table and not shown in heatmap. *X. euvesicatoria* pv. *perforans* strains (rows) are organized by core gene cluster.

We detected 32 predicted type III effector genes in our collection of 270 strains (S5 Fig and S4 Table). The diversity in amino acid sequences of each predicted effector ranged widely from a single conserved allele to 8 or more alleles per gene (Fig 3). None of the effectors were present and intact in 100% of our genomes, partly due to our analysis of draft genomes. The following fourteen effector genes were present in more than 95% of strains and can be considered “core effectors”: *avrBs2*, *xopF1*, *xopF2*, *xopI*, *xopM*, *xopQ*, *xopS*, *xopV*, *xopX*, *xopAE*, *xopAK*, *xopAP*, *xopAU*, and *xopAW*. The genes for *xopD*, *xopE1*, and *xopN* were present in some form in all genomes but more than 5% of strains contained a contig break within the gene. A closer examination of *xopD* by PCR and Sanger sequencing showed this to be an assembly issue due to the repeats within the gene. Effectors at low frequency in our *Xep* strains (<25%) were *xopE3*, *xopAD*, *xopAJ*, *xopAO*, and *xopAQ*. Transcription activator-like (TAL) effectors typically do not assemble in draft genomes due to their characteristic repeat sequences, but there were BLAST hits to previously described TAL effectors in 65 strains. We Sanger sequenced the TAL effector gene in strain 2P6S1 collected in Italy (NCBI accession number OQ588696), which confirmed that it had the same repeat variable diresidues as PthXp1 reported in *Xep* strains from Alabama [50]. The strain isolated in Louisiana, USA was previously reported to have AvrHah1 [16].

The T3E effector XopAF (AvrXv3), which is targeted by the tomato resistance gene *Xv3* [75], was missing or pseudogenized in 64% of strains. Most strains examined from the United States did not have a complete copy of this gene, whereas it was intact in many strains collected in Asia and Africa. The gene for XopJ4 (AvrXv4), recognized by resistance gene *RXopJ4* from *S. pennellii* [76], was present in 88% of strains and absent in all cluster 10 strains and 19 of 88 cluster 2 strains. XopJ2 (AvrBsT), which elicits an HR in pepper but increases virulence in tomato [49], was present in less than half of strains examined (43%) and overwhelmingly in strains from the United States. A homolog of XopJ2, recently designated XopJ2b [77], was present in 50 strains, including two strains from Australia that carried both copies of XopJ2 (S4 Table).

We tested for evidence of positive selection in T3E by estimating synonymous and non-synonymous (dN/dS) substitution rates using a Bayesian approach for detecting pervasive selection (FUBAR, [78]) and maximum likelihood approach for detecting episodic selection (MEME, [79]). We found evidence of pervasive positive selection affecting at least one amino acid in AvrBs2, XopD, XopE1, XopF2, XopK, XopM, XopP and its paralog XopP2, XopQ, XopS, and XopAQ (S4 Table). We found evidence of episodic selection affecting at least one amino acid in XopF2, XopK, XopP, XopP2, XopQ, XopV, and XopAP (S4 Table).

We defined the effector profile of each strain as the predicted presence or absence of each effector and its allelic state, excluding TAL effector hits. Grouping effector profiles according to core gene cluster revealed that allelic variation of effectors often paralleled core genome variation (Fig 3). For example, particular alleles of effectors XopAW, XopQ, and XopP2 were mostly limited to strains in cluster 2. Cluster 3 strains carried unique alleles for effectors XopF2, XopS, XopN, and XopE1, and strains from cluster 7 shared unique alleles for effectors XopF1 and XopZ. Strains from highly diverged cluster 10 had rare alleles in many effectors, and it was the only cluster in which effector XopAJ was found (Fig 3). To visualize variation among strain effector profiles independent of core gene clusters, we transformed dissimilarities between profiles into distances represented in a two-dimensional plot and defined eight effector profile clusters (S6A Fig). Cluster A was characterized by a lack of low frequency effectors and contained 188 strains from 11 of 13 countries (S6 Fig). The remaining effector clusters were defined by the presence of one to three low frequency effectors (S7 Fig). While most effectors were found in multiple countries and continents (S6 Fig), populations in Brazil, Ethiopia, Nigeria, Thailand, South Africa, and the United States contained low frequency effectors that were not widely distributed.

### Copper resistance genes

Xanthomonads, including *Xep*, have acquired genes that confer copper tolerance, likely in response to exposure to copper-based bactericides [33,80–82]. In *Xep*, copper tolerance is conferred by an operon containing the copper resistance genes *copA* and *copB*, and regulator *copL* (*copLAB*) [81]. BLAST analysis showed that these genes were present in 73% of the genomes in our sample (S5 Table). Copper resistance genes are prevalent in the USA; only the genomes from strains isolated from Florida in the early 1990s and a strain from Louisiana lacked *copLAB*. The genes were also missing in the genomes of a few strains from Australia (1), Brazil (2), Ethiopia (2), Mexico (1), and Vietnam (2). In contrast, the genes were absent in all genomes of all strains from Nigeria, China, Iran, Italy, and Thailand.

## Discussion

Emerging plant pathogens have the potential for global outbreaks, exacerbated by complex trade networks. Hybrid tomato production relies on international breeding and production chains with a global network to deliver seeds to growers. Global trade associated with vegetable seed production provides a pathway for global spread of pathogens, with quantities traded that challenge even strong phytosanitary measures [83, 84]. Over 100 countries import seeds of tomatoes and other vegetables; for example, 11.7 million kg of vegetable seed were imported to the USA in 2019, with China being the biggest supplier at 2.4 million kg [85]. *Xanthomonas* species can infest pepper and tomato seed [86], and *Xep* has been isolated from tomato seed [19,87], supporting the hypothesis that seeds can be a source of inoculum for bacterial spot outbreaks [88, 89]. Thirty years after its first report, *Xep* has been identified in tomato production areas around the world [21]. Our results showed extensive genetic diversity in the pathogen, but also genetically similar strains in distant tomato production regions. Furthermore, we found genetically similar strains in seed production and fruit production regions on different continents, as would be expected if the pathogen was being moved in shared production chains. Dated phylogenies indicate multiple waves of diversification of the *Xep* population, before and since its first detection in 1991. Variation in gene content confirms that *Xep* acquired and lost type III effectors during its diversification, which will continue to challenge sustainable management of tomato bacterial leaf spot [49,66].

Using our broad strain collection, we found *Xep* variants in seed production regions in Asia that were previously reported in Australia, Italy, Nigeria, and the United States [43,47,48,57]. Strains from Italy were nearly identical in core genes and very similar in accessory genomes to strains collected from Thailand and Vietnam (cluster 7), both major seed production regions. The atypical bacterial spot strains from Nigeria, recently designated as race T5 [24], were genetically similar to strains from Thailand (cluster 10). Cluster 10 strains may encompass one or more new subspecific taxa within *X. euvesicatoria*. A recently described variant of *Xep* in Florida [cluster 3; [43,47]], which was also found in Australia [57], was similar in the core genome to strains found in China (cluster 3); however, these strains showed divergence in the pangenome, consistent with accessory genome evolution in emergent populations. Beyond previously described variants, we found strains in Iran that were closely related to strains from China (cluster 9); multiple instances of genetic similarity between strains from North America and Ethiopia (clusters 1 and 4); and highly similar strains shared between USA and Brazil (cluster 8), USA and Australia (clusters 1 and 3), and between Australia and Vietnam (cluster 1). Given the variation of *Xep* across our sample, genetic similarity in core genes and gene content across continents is strong evidence of international dissemination. Genetically similar strains of bacterial spot pathogens *X. euvesicatoria* pv. *euvesicatoria* and *X. hortorum* pv. *gardneri* collected from different continents similarly suggest intercontinental dissemination in tomato and pepper seed [15,58,90]. Whole genome analysis of *X. hortorum* pv. *pelargonii* strains from a 2022 epidemic of bacterial blight of geranium in the USA showed zero to seven chromosomal SNPs among isolates of the emergent strain that was distributed to multiple states in plant cuttings [9,91].

Other *Xep* genotypes indicated a more limited distribution. We did not find core gene cluster 2 strains in the seed production regions sampled (China, Thailand, Vietnam), while this lineage was highly represented in our USA sample. To date, strains in this cluster have been found only in the southeastern and midwestern USA [43,47,50,53,54] and Mexico. Seedling nurseries in the southeast USA produce tomato transplants for growers in multiple states. Interstate movement of strains on seedlings is likely responsible, at least in part, for disseminating genetically similar strains to different states [43,53]. We previously reported extensive recombination with *X. euvesicatoria* pv. *euvesicatoria* in cluster 2 strains [47] and this cluster had a diverse accessory genome. Additional research is needed to determine the population dynamics and genetic mechanisms that underly the diversity in this cluster.

The *Xep* strains we examined from the USA were assigned to core gene clusters 1, 2, 3, 4, 5, 6, and 8, representing several distinct genetic lineages. The US sample had an overall negative Tajima’s D value. A negative value of Tajima’s D across the USA sample suggests an abundance of low frequency haplotypes, which is consistent with low frequency clonal lineages or accumulation of mutations within lineages. Tajima’s D varied from positive to negative within individual states and in samples from other countries, although values for small sample sizes should be interpreted with caution.

To better understand the initial emergence of *Xep*, we used calibrated phylogenies to examine the timing of lineage divergence. International trade in F1 hybrid tomato seed surged in the second half of the 20^th^ century, after the first hybrid tomato cultivars were released by 1940 [92, 93]. There was a 300-fold increase in hybrid tomato seeds exported from Asia between 1962 and 1977 [94, 95] and subsequent rapid growth in tomato production. Our analyses estimate the most recent common ancestor of our sample to ~150 years ago, while the major ancestral lineages diverged during or after the early expansion in the hybrid seed trade. We hypothesize that the emergence and geographic distribution of lineages may be associated with the multinational structure of tomato breeding and seed production, in which parental lines and geographic locations of seed production change over time [96].

Bacterial spot is a destructive disease in areas where tomatoes are grown under humid conditions and growers in the USA have relied heavily on copper bactericides to manage this disease. In response, *Xep* strains have developed copper tolerance [26]. Most strains isolated from Florida in the 1990s lacked the *copLAB* genes, but they are now common in strains collected in the USA [97]. Recent studies of Florida strains found that these copper resistance genes are more frequently present on the chromosome than on a plasmid, suggesting selection for vertical inheritance of copper tolerance [33,97]. In contrast, strains from other countries lacked copper resistance genes, indicating little or no local selection for the acquisition of *cop* genes.

Type III effectors are important members of *Xanthomonas* genomes given their roles in pathogenicity and virulence. We found up to 16 putative core effector genes, most of which exhibited allelic variation. The impact of allelic variation in *Xep* effectors on pathogen fitness, if any, is unknown. Signatures of positive selection on some genes indicate past or ongoing fitness impacts. Low frequency effectors were found across core gene clusters, suggesting acquisition of new effectors and their exchange among *Xep* lineages. For example, some strains in clusters 3, 4, and 5 from the United States, Canada, and Mexico carried the same alleles of low frequency effectors XopAQ and XopE3 as strains from Asia, Nigeria, and Italy. BLAST analysis suggested the geographically widespread presence of transcription activation-like (TAL) effectors in *Xep*. Both TAL effectors described in *Xep*, *avrHah1* and *pthXp1*, are associated with increased disease severity on tomato [16,50]. Acquisition of T3Es could increase the fitness of *Xep* relative to other bacterial spot pathogens and cause more damaging disease outbreaks [49,51,66].

The release of new plant varieties that carry disease resistance genes can have dramatic effects on pathogen population structure due to selection to overcome host resistance [98–100], and we have previously reported on the loss of function of effector AvrXv3 (XopAF) across lineages [30,53,66]. Examination of T3E content at a global scale puts variation previously observed in Florida into a larger context. XopAF was present in strains collected in the 1990s (cluster 1), but absent or non-functional in most strains from Florida, Indiana Ohio, and North Carolina, USA [26,28,30,53,66]. Here, we found that *xopAF* was intact in many strains from seed production areas, but confirmed that it is no longer a viable target for resistance in the USA. The effector XopJ4 is a potential resistance target [66] based on its recognition by *RXopJ4* from *S. pennellii* [76], but Klein-Gordon et al. [26] reported that it was missing from 3.2% of Florida strains collected in 2017 and, here, we found that it was absent in one North Carolina and 20 Florida, USA strains. All strains collected outside the USA contained *xopJ4*, except for cluster 10 strains. Another XopJ family member, *xopJ2*, is a virulence factor in tomato [49,51]. This T3E is common in North America, particularly in cluster 2 and 6 strains, but absent or infrequently detected in *Xep* populations elsewhere. An alternative form of this effector, recently described as XopJ2b [77], is more common in strains from outside North America. Resistance to strains with XopJ2 (XopJ2a) is conferred by Ptr-1 from the wild relative of tomato *Solanum lycopersicoides* [101], which may also be effective against XopJ2b [77]. Alternative resistance strategies include transgenic *Bs2* [59] and CRISPR editing of the *bs5* homologs in tomato [102], which are expected to provide resistance to many or all *X. perforans* strains, respectively.

In summary, we found strong evidence for intercontinental movement of *Xep*, consistent with the international nature of tomato breeding and hybrid tomato seed production. We also found notable diversity in our global sample of *Xep*, including in seed production regions, and multiple variants of *Xep* that do not appear to be widely distributed. Our results also suggest that continued monitoring of bacterial spot pathogens is warranted to identify emerging lineages that may respond differently to disease management with copper-based products and effector-targeted host resistance. These findings also raise questions regarding the degree of genetic variation seen in *Xep*, such as the evolutionary genetic mechanisms that are responsible and the effects of different genetic variants on epidemiology. The genomic diversity of *Xep* in seed and fruit production regions creates the opportunity for recombination among strains and dissemination of high fitness variants of *Xep*.

## Materials and methods

### Bacterial strains, genome sequencing, and assembly

*Xep* strains were collected from 13 different countries (Tables 1 and S1). Strains from the United States were collected from seven states between 1991 to 2016 and comprised 181 strains. The remaining 89 strains were collected from Canada, Mexico, Brazil, Italy, Ethiopia, Nigeria, South Africa, Iran, China, Thailand, Australia, and Vietnam. Strains from China, Thailand, and Vietnam were collected from fields designated for production of tomato seed for the global market. Strains from Brazil were obtained from both staked fresh-market and processing tomato commercial fields. Strains from Italy were isolated from tomato pith in greenhouse tomato showing wilting symptoms [64,103]. Strains from South Africa were collected from commercial seed lots. Strains from Nigeria were obtained from fields cultivated for both subsistence and commercial purposes. Strains from other countries were collected from fields designated for commercial fruit production.

A total of 270 *Xep* genome sequences were used during this study (S1 Table). Draft and whole genomes of 117 strains were generated and published previously [43,47,48,57,58,67,103]. The remaining 153 strains were sequenced for this study using Illumina platforms. Genomic DNA was extracted from single colony cultures grown for 24-hr in nutrient broth using the Wizard Genomic DNA Purification Kit (Promega, Chicago, IL) following manufacturer instructions. Genomic libraries for sequencing were prepared using the Nextera DNA library preparation kit from Illumina (Illumina, San Diego, CA). Sequencing was performed at the Interdisciplinary Center for Biotechnology Research, University of Florida, using an Illumina MiSeq to generate 250 bp paired end reads for each strain. Additional genomic sequence data were generated for five strains for the ANI analysis (S1B Table). Genomic DNA was extracted using the above methods except that extracted genomic DNA was sent to SeqCenter (Pittsburg, PA) for sequencing with Illumina NovaSeq 6000, producing 150 bp paired end reads.

Raw reads were trimmed of adapters and paired with Trim Galore (https://github.com/FelixKrueger/TrimGalore) [104], then assembled into contigs with Spades version 3.10.1 [105], with k-mers 21, 33, 55, 77, 99, and 127 with read error correction and “--careful” switch. Reads were then aligned to the assembled contigs using Bowtie 2 v. 2.3.3 [106]. Inconsistencies were identified and polished using Pilon [107]. Contigs smaller than 500 bp and with less than 2.0 k-mer coverage were filtered out. Quality of genomes was assessed with CheckM [108]. Assembled genomes were annotated using the IMG/JGI platform [109]. The genome data generated for this study are available in NCBI BioProject PRJNA941448.

### Core gene phylogeny

In a previous study, we defined a set of 1,356 ‘core genes’ from 58 genomes of *Xep* strains isolated from Florida [47]. The core genes were determined based on amino acid sequence homology using GET_HOMOLOGUES software package [110]. We used the core genes from a representative *Xep* genome, Xp91-118, as query to search the remaining 269 genomes using local BLAST [111]. BLAST results were filtered using query coverage and pairwise nucleotide sequence alignment thresholds of 70% each and the sequence was checked for the presence of standard start and stop codons at either end of the gene and gene was removed if both were not present. A total of 887 genes were found to be intact in all 270 genomes. Genes were individually parsed and aligned using MAFFT [112] and concatenated using sequence matrix [113]. The result was a 617.854 Kbp alignment, hereafter referred to as core genes.

The concatenated core gene sequence was used to construct a maximum likelihood (ML) phylogenetic tree using RAxML v.8.2.12 [114]. General time reversible model with gamma distributed rates and invariant sites (GTRGAMMA) was used as the nucleotide substitution model. To account for recombination, the ML tree output from RAxML and concatenated core genome alignment were used as input for ClonalFrameML v1.12 [71].

### Population structure

SNPs were extracted from core genes for hierarchical clustering based on Bayesian analysis of population structure (hierBAPS) algorithm [115], implemented in the ‘rhierBAPS’ R package v 1.0.1 [72,116]. For visualization, hierBAPS clusters were added to the phylogenetic tree generated from ClonalFrameML using the ‘ggtree’ package in R [117]. The treemap function in plotly [118] was used to show the relative distribution of clusters across geographic locations. R package ‘ggplot2’ was used to map hierBAPS clusters to countries [119]. The ‘PopGenome’ R package [120] was used to calculate FST, Watterson’s theta, nucleotide diversity (pi) [69], and Tajima’s D statistic [70] by geographic location and by hierBAPS cluster.

Assembled genomes were used for calculating average nucleotide identity and pangenome analysis. Average nucleotide identity (ANIb) between strains was calculated using whole genome assemblies with Pyani version 0.2.10 [121]. The pangenome was estimated using Roary v3.12.0 [122] after annotation from Prokka v1.12 [123]. The gene presence absence matrix from Roary (S4 Data) was used as input for generation of NMDS plots using the ‘dplyr’ and ‘ggplot2’ packages from tidyverse [119] and to generate gene accumulation curves for each cluster using package ‘micropan’ [124].

### Bayesian analysis of *X. euvesicatoria* pv. *perforans* divergence times

A whole genome alignment was generated using split k-mer analysis version 2 (SKA2) [125] for all 270 *Xep* strains plus outgroup *X. euvesicatoria* pv. *euvesicatoria* strain 85-10 (NCBI Accession GCA_000009165.1; S2 Data). The alignment was reduced to variable sites only using Geneious 2023.2.1 (BioMatters Ltd.). A phylogenetic network was calculated from the resulting SNPs using the NeighborNet 2004 algorithm in SplitsTree5 [126,127]. Phylogenetic conflict was indicated between the 259 strains, cluster 10 strains, and outgroup (S1B Fig). Removing the cluster 10 strains did not remove the phylogenetic conflict (reticulations) between *Xep* and *Xee* outgroup. Because the location of the root was not clear, we limited our dating of the phylogeny of *Xep* to the 259 strains in BAPS clusters 1 through 9 and inferred the root position as part of the analysis. We used Gubbins v. 2.4.1 [73] to remove putative recombinant sites from whole genome alignments generated using SKA2 [125] and the complete genome of Xp91-118 as a reference (GCF_000192045.2). The resulting alignment was used to infer a phylogenetic tree using the GTRGAMMI model in RAxML version 8.2.10 [128]. The temporal analysis was conducted with BactDating v1.1.1 [74]. The inputs to the BactDating analysis were the maximum likelihood tree and dates of isolation assigned as dates of tips. The rooting of the tree was estimated using the initRoot function, which maximizes the correlation between tip date, the year the strain was collected, and root-to-tip branch lengths. Dates of nodes were inferred using the bactdate function on the re-rooted tree using a relaxed molecular clock with Markov chain Monte Carlo (MCMC) chains of 10^6^ iterations. Phylostems [129] was used to assess the temporal signals within internal clades for interpretation of node date inferences.

We also used BEAST v. 1.10.4 [130] to infer a dated phylogeny. The XML file was manually edited to include the ‘ascertained’ flag in the alignment block (S3 Data). The HKY nucleotide substitution model with empirical base frequencies and gamma distribution of site-specific rate heterogeneity was used with coalescent Bayesian skyline priors with an uncorrelated relaxed clock for Bayesian phylogenetic inference over MCMC chains of 200 million generations. Adequate mixing was assessed based on a minimum effective sample size of 200 for parameter estimates as calculated by Tracer v. 1.10.4. A maximum clade credibility tree was inferred from the posterior distribution of trees using TreeAnnotator v. 1.10.4, specifying a burn-in of 10% and the ‘keep’ option for node heights. Trees were visualized in iTOL version 6.9.1 [131].

### Type III effector analysis

A T3E database was generated using amino acid sequences of 63 *Xanthomonas* effectors based on a community-curated list [132] (S6 Table). When available, functional annotations were retrieved from NCBI and Pfam databases [133]. Orthologous sequences were identified with the software BLASTp [134,135], by querying the curated effectors database against the amino acid sequences of the annotated genomes of 270 *Xep* strains. Sequences (BLAST hits) were considered effector orthologs when at or above a threshold of 70 percent identity and 50 percent query coverage. When multiple sequences from the same strain had hits above the thresholds to a particular effector, we used the product of the percent identity and query coverage to select the best overall hit. Sequences with homology to multiple effectors and sequences with evidence of contig breaks were manually removed. Assignment of sequences as effector orthologues was confirmed by performing a clustering analysis of all sequences using the software USEARCH v. 11.0.667 and the algorithm HPC-CLUST [136]. For the duplicated effector XopP, we used a phylogenetic analysis of all sequences to distinguish likely orthologous alleles from the more genetically distant paralogous sequences, which were assigned to XopP2.

Orthologous sequences from each effector were extracted from the annotated genomes, aligned with MAFFT [112], and allelic variants identified [137] to generate a numeric matrix representing presence and allelic variant or absence. Hierarchical clustering analysis of effectors was performed by calculating a distance matrix with function ‘dist’ with the method ‘manhattan’, and the function ‘hclust’ with the method ‘complete’ from the R package ‘vegan’ [116,138]. The results were displayed as a heatmap with the package ‘gplots’ and the function heatmap.2 [139].

To investigate the presence of positive selection acting on the effector sequences, we used the software HyPhy (Hypothesis Testing using Phylogenies) implementing the methods FUBAR (Fast, Unconstrained Bayesian AprRoximation) and MEME (Mixed Effects Model of Evolution) [78, 79]. The Bayesian method FUBAR evaluates pervasive selection, assuming the same rates of synonymous and nonsynonymous substitution per site on all branches. The method MEME uses a maximum likelihood approach to evaluate episodic selection, i.e., selection only a subset of branches of the phylogeny. For each effector gene, a codon-aware alignment was generated with the software PRANK using the codon flag ‘-c’ as settings [140]. RAxML [114] was used to infer a phylogenetic tree with the GTRGAMMA (gamma time-reversible) model of nucleotide substitution. The codon-aware alignment and phylogenetic tree were used as the input files for FUBAR and MEME.

To determine the relationship of the effector profiles with respect to core gene cluster, geographic and temporal distribution, we transformed the dissimilarities in the matrix of effector profiles into distances with non-metric multidimensional scaling (NMDS). We used the Bray-Curtis dissimilarity index, a robust index able to handle missing data that considers the presence and absence of effectors as equally informative, calculated with the package ‘vegan’ and the function ‘metaMDS’ [138]. We used a low number of dimensions (K=2) and set try=30 and trymax=500 for random starts to avoid the NMDS getting trapped in local optima. NMDS plots were created with the packages ‘ggrepel’ and ‘ggplot2’ [141,142]. Based on the NMDS analysis, we assigned strains to effector clusters, which were plotted on a worldwide map with the packages ‘ggplot2’ and ‘scatterpie’ [142,143]. The map was created in R with the packages ‘cowplot’, ‘ggrepel’, ‘ggspatial’, ‘libwgeom’, ‘sf’, ‘rgeos’, ‘memisc’, ‘oz’, ‘maptools’ and ‘rnaturalearth’ with the function ‘ne_countries’ [141,144–151]. Geographic coordinates (longitude, latitude) of countries and states (for USA) of collection were obtained with the R package ‘googleway’ [152] and the function ‘mutate_geocode’ from Google maps.

To sequence the putative TAL effector from 2P6S1, native plasmid DNA was isolated using the alkaline lysis method [153]. *Eco*RI digested DNA of the plasmid prep was ligated into vector pLAFR3 [154] restricted with the same enzyme for transformation into *E*. *coli* DH5α. Clones containing the TAL effector were identified by PCR and analyzed by restriction digest. One clone, designated as p7.1, contained an approx. 5 Kbp *Eco*RI fragment and was selected for Sanger sequencing and phenotype testing. For Sanger sequencing of the TAL repeat region, DNA of p7.1 was restricted with *Nsi*I and the internal fragment was ligated into vector pBluescript restricted with *Pst*I. Additional pBluescript subclones were made using *Bam*HI (~3 Kbp and ~1.1 Kbp) and *Bam*HI/*Eco*RI (~1 Kbp) in order to cover the entire cloned region in p7.1. All clones were transformed into DH5α for sequencing using vector primers T3 and T7.

Copper resistance genes in assembled genomes were identified with BLASTn analysis using *copL* (MBZ2440241.1), *copA* (MBZ2440240.1), and *copB* (MBZ2440239.1) from *Xep* strain Xp2010 as reference sequences [33].

## Supporting information

S1 Fig Phylogenetic analysis of 270 *X. euvesicatoria* pv. *perforans* strains. (A) Maximum likelihood phylogenetic tree of *Xanthomonas euvesicatoria* pv. *perforans* strains based on aligned nucleotide sequences of 887 core genes, also used for Fig 1A. The tree was inferred using RAxML using a GTRGAMMAI substitution model. The tree was rooted using the 11 genetically diverged strains that make up core gene cluster 10. (B) NeighborNet network inferred using SNPs from aligned whole genome sequences, including *X. euvesicatoria* pv. *euvesicatoria* strain 85-10 (bolded) as an outgroup. Core gene cluster 10 strains are highlighted. Reticulations in the network indicate conflicting phylogenetic relationships.
(PDF)

S2 Fig Accessory genome variation in *Xanthomonas euvesicatoria* pv. *perforans*. (A) Visualization of pangenome variation by non-metric multidimensional scaling of gene presence-absence for all 270 *X. perforans* strains by BAPS cluster. Ellipses assume a multivariate t-distribution. (C) Increase in gene count with increasing number of strains sampled. Clusters 1 and 2 were represented by the most strains, but other clusters showed similar rates of increase in the pangenome of the cluster. Pangenome matrix used for analysis is available as S4 Data.
(PDF)

S3 Fig Temporal signal in phylogenetic tree of 259 *X. euvesicatoria* pv. *perforans* strains. (A) Correlation between sampling year and root-to-tip distance in maximum likelihood phylogenetic tree inferred from alignment of whole genome sequences. Output was generated from BactDating R package. (B) Temporal signal within the phylogenetic determined using Phylostems tool. Nodes with significant temporal signals are indicated with colored circles. Adjusted R-squared values by color are: dark green 0–0.2; light green 0.2–0.4; yellow 0.4–0.6; orange 0.6–0.8; red 0.8–1.
(PDF)

S4 Fig Dated phylogenies of 259 *X. euvesicatoria* pv. *perforans* strains. (A) Dating using BactDating relaxed clock analysis on RAxML-generated phylogeny. This is the tree shown in Fig 2, shown here without collapsed nodes. (B) Dating of same dataset using BEAST with coalescent Bayesian skyline priors and an uncorrelated relaxed clock.
(PDF)

S5 Fig Frequency of Xop effectors among 270 *Xanthomonas euvesicatoria* pv. *perforans* strains. The most common allele observed was assigned to allele type 1, second most frequent allele to allele type 2, and so on. Note that alleles classified as pseudogenes included contig breaks, which include assembly errors. For example, all strains appear to have *xopD*, but a repeat caused a contig break in the gene in nearly half of the genomes.
(PDF)

S6 Fig Clustering of 270 *Xanthomonas perforans* effector profiles by non-metric multidimensional scaling and distribution of resulting clusters among geographic regions. Analysis did not include TAL effectors. (A) The most frequently observed group of effector profiles form cluster A. This cluster of 188 strains is represented as a star in plots B-C, as it is represented in most BAPS core gene clusters (B), most of the sampled tomato production regions (C), and in collections from 1991 to 2017 (D). Clusters were largely defined by low frequency effectors (S7 Fig). (E) Distribution of strains by effector clusters among sampled countries. Base layer of map is from Natural Earth (https://www.naturalearthdata.com).
(PDF)

S7 Fig Variation in type III effector profiles in 270 *Xanthomonas euvesicatoria* pv. *perforans* strains ordered according to NMDS of effector profiles. Analysis did not include TAL effectors. Type III effectors are in columns and *Xep* strains in rows. Effector status is shown by allele type: absence is indicated by allele type 0 (white), while the most frequent allele observed when the effector is present is allele type 1 (purple), second most frequent is allele type 2 (blue), and so on. Putative pseudogenized effectors are shown as allele 13 (gray). The order of columns was determined by hierarchical clustering analysis, placing similarly distributed effectors adjacent to each other. Order of rows is based on NMDS clustering analysis of effector profiles (see S6 Fig).
(PDF)

S1 Table Genome data and metadata for *X. euvesicatoria* pv. *perforans* strains. (A) BAPS cluster assignment for each strain, NCBI Accession for each genome, and associated genome assembly statistics. (B) Additional genomic data used only for ANI comparisons in Fig 1D.
(XLSX)

S2 Table Genetic diversity statistics by geographic region and BAPS group. For each geographic region and BAPS group, we calculated: the number of SNPs in the 617854 bp alignment; Watterson’s Theta per site; Pi, the average number of differences per site; and Tajima’s D. (A) Statistics by country and U.S. state. (B) Statistics by BAPS group.
(XLSX)

S3 Table Average nucleotide identity (ANIb) comparisons between strains with highly similar core gene sequences collected across continents. (A) Proportion nucleotide identity. (B) Alignment fraction.
(XLSX)

S4 Table Putative type III effectors (Xop proteins) found in 270 *X. euvesicatoria* pv. *perforans* assembled genomes. (A) Summary for each locus. (B) Results by strain. Each different amino acid sequence per gene was assigned a numerical allele type, such that the most common allele observed was assigned to allele type 1. Potential pseudogenes are indicated with “pseudo” and absence indicated with zero. Locus tags refer to JGI IMG annotations (https://img.jgi.doe.gov). Reference sequences used for BLAST searches are given in S5 Table. The final column shows the result of BLAST searches for TAL effectors.
(XLSX)

S5 Table Presence or absence of copper genes (*copLAB*) in 270 *X. euvesicatoria* pv. *perforans* assembled genomes. Symbols represent gene presence ‘+’ or absence ‘-’. Contig break in gene is indicated by (+).
(XLSX)

S6 Table Type III effector database used to query assembled genomes for effector genes. (XLSX)

S1 Data Nucleotide alignment of 887 core genes from 270 *X. euvesicatoria* pv. *perforans* strains. Alignment is 617,855 bp in FASTA format.
(ZIP)

S2 Data Nucleotide alignment of variable sites from whole genome alignment of 270 X. *euvesicatoria* pv. *perforans* strains and *X. euvesicatoria* pv. *euvesicatoria* strains 85-10.
(ZIP)

S3 Data XML file used for BEAST analysis. (ZIP)

S4 Data Pangenome matrix for 270 *X. euvesicatoria* pv. *perforans* strains. (ZIP)
