# Supplementary material for: Diversification of an emerging bacterial plant pathogen; insights into the global spread of Xanthomonas euvesicatoria pv. perforans
Source: PLoS Pathog. 2025 Apr 9;21(4):e1013036. doi: 10.1371/journal.ppat.1013036 (PMC12047805; doi:10.1371/journal.ppat.1013036)
Supplement: S1 Fig — (A) Maximum likelihood phylogenetic tree of Xanthomonas euvesicatoria pv. perforans strains based on aligned nucleotide sequences of 887 core genes, also used for Fig 1A. The tree was inferred using RAxML using a GTRGAMMAI substitution model. The tree was rooted using the 11 genetically diverged strains that make up core gene cluster 10. (B) NeighborNet network inferred using SNPs from aligned whole genome sequences, including X. euvesicatoria pv. euvesicatoria strain 85-10 (bolded) as an outgroup. Core gene cluster 10 strains are highlighted. Reticulations in the network indicate conflicting phylogenetic relationships. (PDF) [file ppat.1013036.s001.pdf]

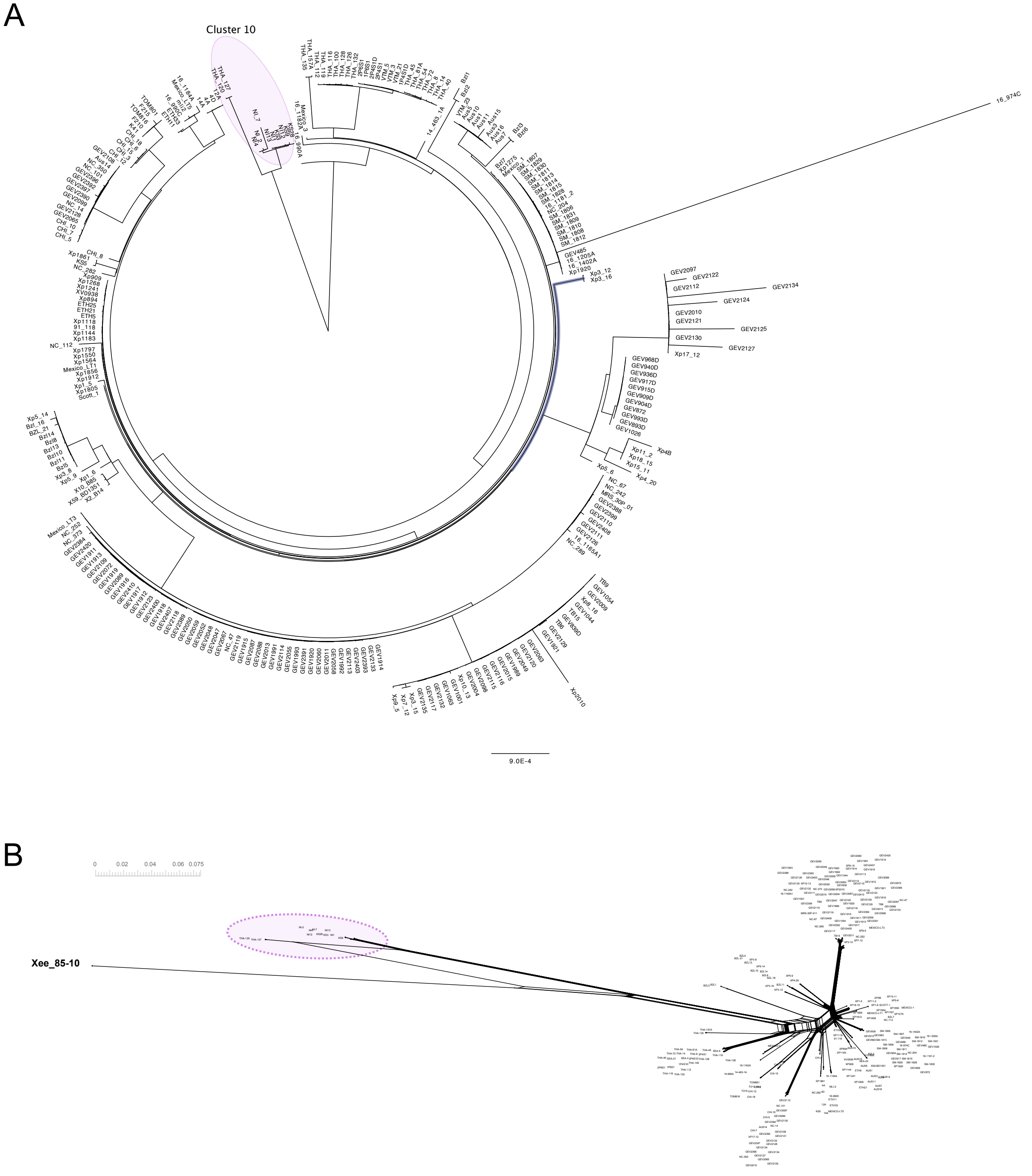

S1 Figure. Phylogenetic analysis of 270 *X. euvesicatoria* pv. *perforans* strains. (A) Maximum likelihood phylogenetic tree of *Xanthomonas euvesicatoria* pv. *perforans* strains based on aligned nucleotide sequences of 887 core genes. The tree was inferred using RAxML using a GTRGAMMAI substitution model. The tree was rooted using the 11 genetically diverged strains that make up core gene cluster 10. (B) Neighbor-net network inferred using SNPs from aligned whole genome sequences, including *X. euvesicatoria* pv. *euvesicatoria* strain 85-10 (bolded) as an outgroup. Core gene cluster 10 strains are highlighted. Reticulations in the network indicate conflicting phylogenetic relationships.
