# Supplementary material for: Diversification of an emerging bacterial plant pathogen; insights into the global spread of Xanthomonas euvesicatoria pv. perforans
Source: PLoS Pathog. 2025 Apr 9;21(4):e1013036. doi: 10.1371/journal.ppat.1013036 (PMC12047805; doi:10.1371/journal.ppat.1013036)
Supplement: S2 Fig — (A) Visualization of pangenome variation by non-metric multidimensional scaling of gene presence-absence for all 270 X. perforans strains by BAPS cluster. Ellipses assume a multivariate t-distribution. (C) Increase in gene count with increasing number of strains sampled. Clusters 1 and 2 were represented by the most strains, but other clusters showed similar rates of increase in the pangenome of the cluster. Pangenome matrix used for analysis is available as S4 Data. (PDF) [file ppat.1013036.s002.pdf]

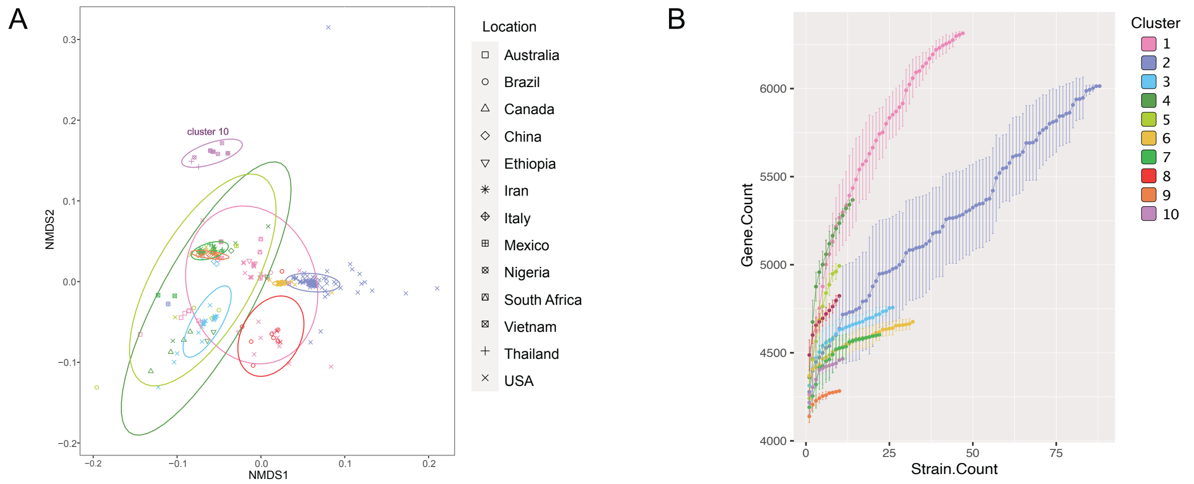

S2 Figure. Accessory genome variation in *Xanthomonas euvesicatoria* pv. *perforans*. (A) Visualization of pangenome variation by non-metric multidimensional scaling of gene presence-absence for all 270 *X. perforans* strains by BAPS cluster. Ellipses assume a multivariate t-distribution. (B) Increase in gene count with increasing number of strains sampled. Clusters 1 and 2 were represented by the most strains, but other clusters showed similar rates of increase in the pangenome of the cluster.
