# Supplementary material for: Diversification of an emerging bacterial plant pathogen; insights into the global spread of Xanthomonas euvesicatoria pv. perforans
Source: PLoS Pathog. 2025 Apr 9;21(4):e1013036. doi: 10.1371/journal.ppat.1013036 (PMC12047805; doi:10.1371/journal.ppat.1013036)
Supplement: S3 Fig — (A) Correlation between sampling year and root-to-tip distance in maximum likelihood phylogenetic tree inferred from alignment of whole genome sequences. Output was generated from BactDating R package. (B) Temporal signal within the phylogenetic determined using Phylostems tool. Nodes with significant temporal signals are indicated with colored circles. Adjusted R-squared values by color are: dark green 0–0.2; light green 0.2–0.4; yellow 0.4–0.6; orange 0.6–0.8; red 0.8–1. (PDF) [file ppat.1013036.s003.pdf]

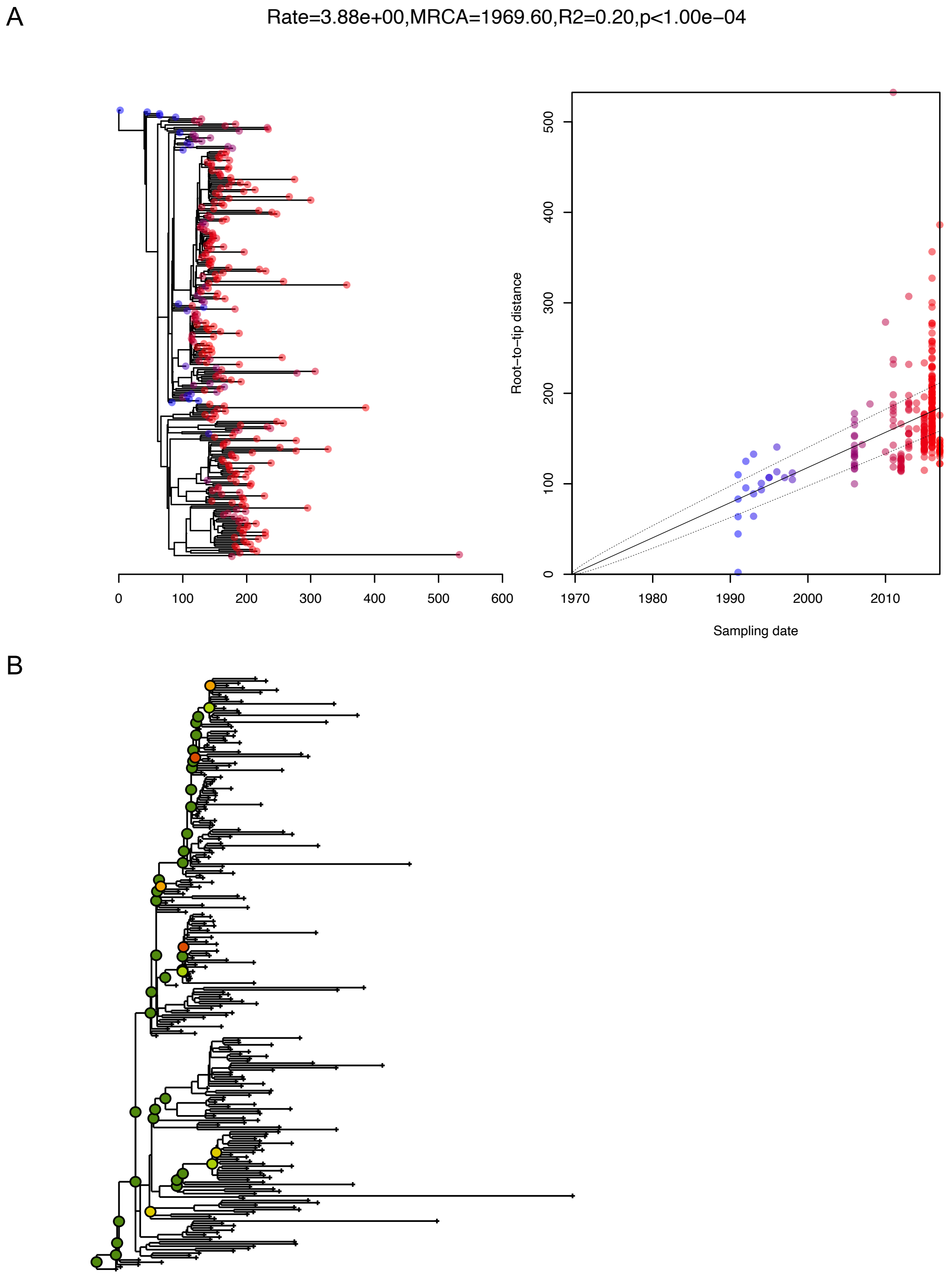

S3 Figure. Temporal signal in phylogenetic tree of 259 *X. euvesicatoria* pv. *perforans* strains. (A) Correlation between sampling year and root-to-tip distance in maximum likelihood phylogenetic tree inferred from alignment of whole genome sequences. Output was generated from BactDating R package. (B) Temporal signal within the phylogenetic determined using Phylostems tool. Tree is rooted as in Figure 2. Nodes with statistically significant temporal signals are indicated with colored circles. Adjusted R-squared values by color: dark green 0–0.2; light green 0.2–0.4; yellow 0.4–0.6; orange 0.6–0.8; red 0.8–1.
