# Supplementary material for: Diversification of an emerging bacterial plant pathogen; insights into the global spread of Xanthomonas euvesicatoria pv. perforans
Source: PLoS Pathog. 2025 Apr 9;21(4):e1013036. doi: 10.1371/journal.ppat.1013036 (PMC12047805; doi:10.1371/journal.ppat.1013036)
Supplement: S4 Fig — (A) Dating using BactDating relaxed clock analysis on RAxML-generated phylogeny. This is the tree shown in Fig 2, shown here without collapsed nodes. (B) Dating of same dataset using BEAST with coalescent Bayesian skyline priors and an uncorrelated relaxed clock. (PDF) [file ppat.1013036.s004.pdf]

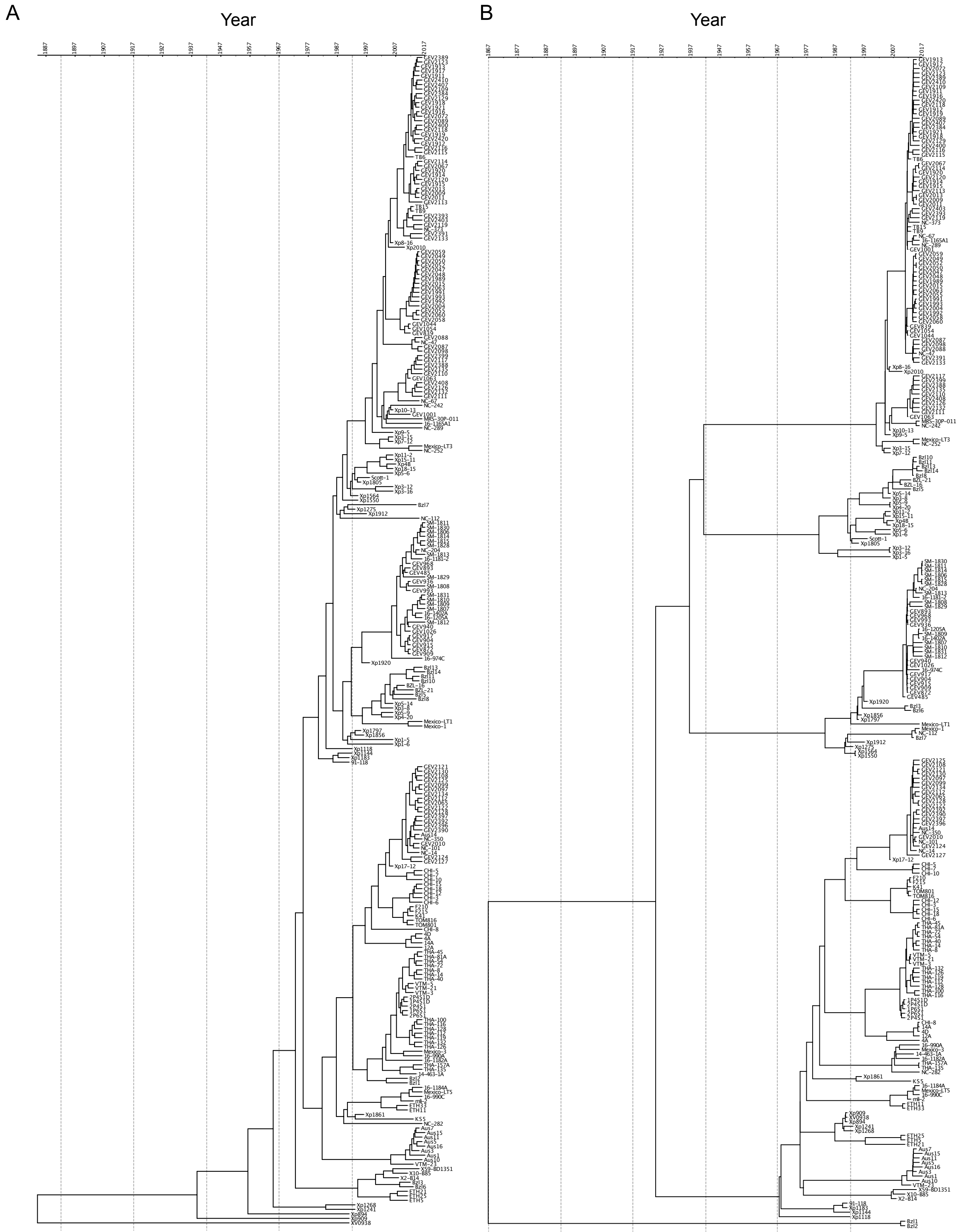

S4 Figure. Dated phylogenies of 259 *X. euvesicatoria* pv. *perforans* strains. (A) Dating using BactDating relaxed clock analysis on RAxML-generated phylogeny. This is the tree shown in Figure 2, shown here without collapsed nodes. (B) Dating of same dataset using BEAST with coalescent Bayesian skyline priors and an uncorrelated relaxed clock.
