# Supplementary material for: Diversification of an emerging bacterial plant pathogen; insights into the global spread of Xanthomonas euvesicatoria pv. perforans
Source: PLoS Pathog. 2025 Apr 9;21(4):e1013036. doi: 10.1371/journal.ppat.1013036 (PMC12047805; doi:10.1371/journal.ppat.1013036)
Supplement: S5 Fig — The most common allele observed was assigned to allele type 1, second most frequent allele to allele type 2, and so on. Note that alleles classified as pseudogenes included contig breaks, which include assembly errors. For example, all strains appear to have xopD, but a repeat caused a contig break in the gene in nearly half of the genomes. (PDF) [file ppat.1013036.s005.pdf]

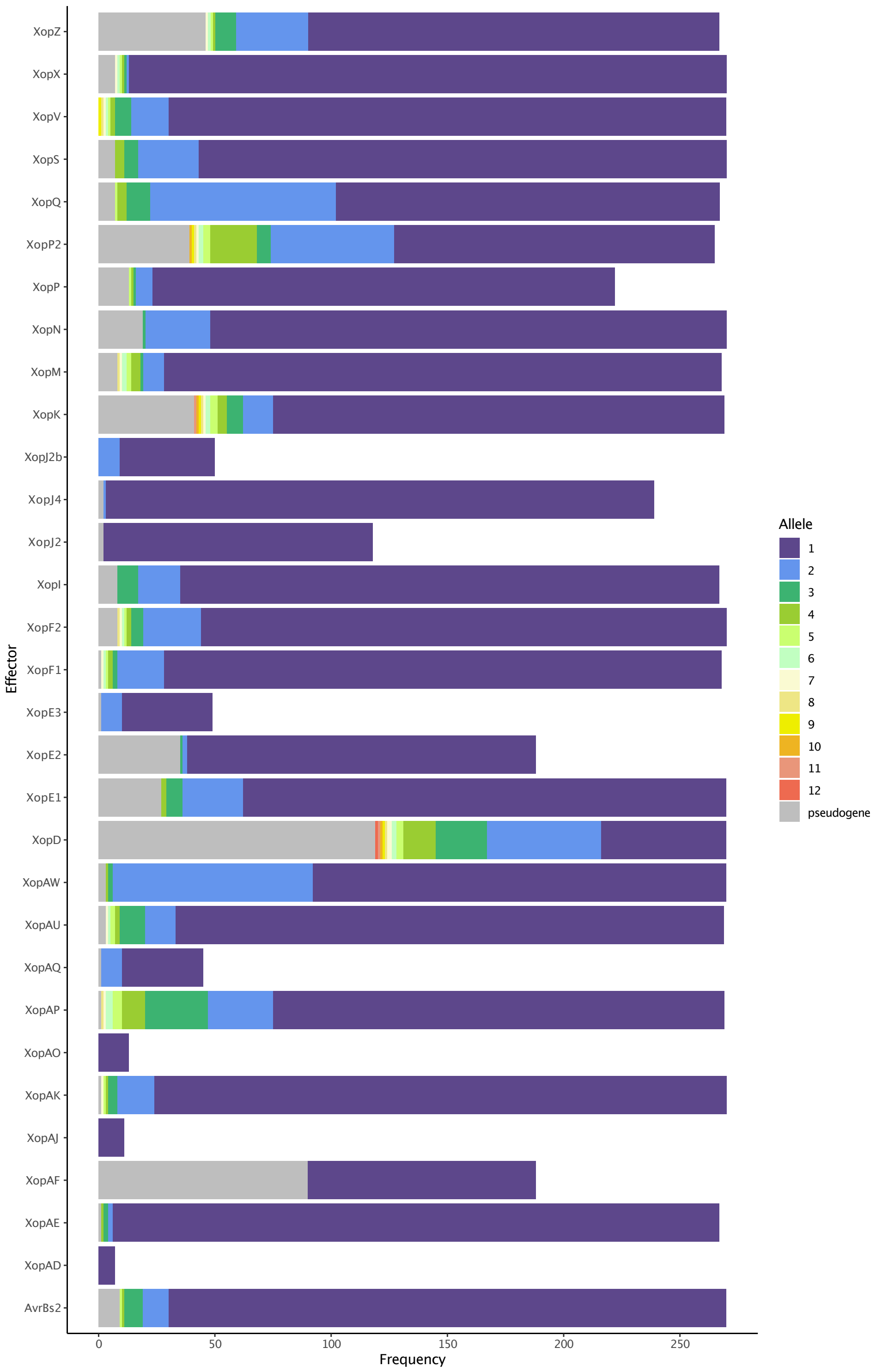

S5 Figure. Frequency of Xop effectors among 270 *Xanthomonas euvesicatoria* pv. *perforans* strains. The most common allele observed was assigned to allele type 1, second most frequent allele to allele type 2, and so on. Note that alleles classified as pseudogenes included contig breaks, which include assembly errors. For example, all strains appear to have *xopD*, but a repeat caused a contig break in the gene in nearly half of the genomes.
