# Supplementary material for: Diversification of an emerging bacterial plant pathogen; insights into the global spread of Xanthomonas euvesicatoria pv. perforans
Source: PLoS Pathog. 2025 Apr 9;21(4):e1013036. doi: 10.1371/journal.ppat.1013036 (PMC12047805; doi:10.1371/journal.ppat.1013036)
Supplement: S6 Fig — Analysis did not include TAL effectors. (A) The most frequently observed group of effector profiles form cluster A. This cluster of 188 strains is represented as a star in plots B-C, as it is represented in most BAPS core gene clusters (B), most of the sampled tomato production regions (C), and in collections from 1991 to 2017 (D). Clusters were largely defined by low frequency effectors (S7 Fig). (E) Distribution of strains by effector clusters among sampled countries. Base layer of map is from Natural Earth (https://www.naturalearthdata.com). (PDF) [file ppat.1013036.s006.pdf]

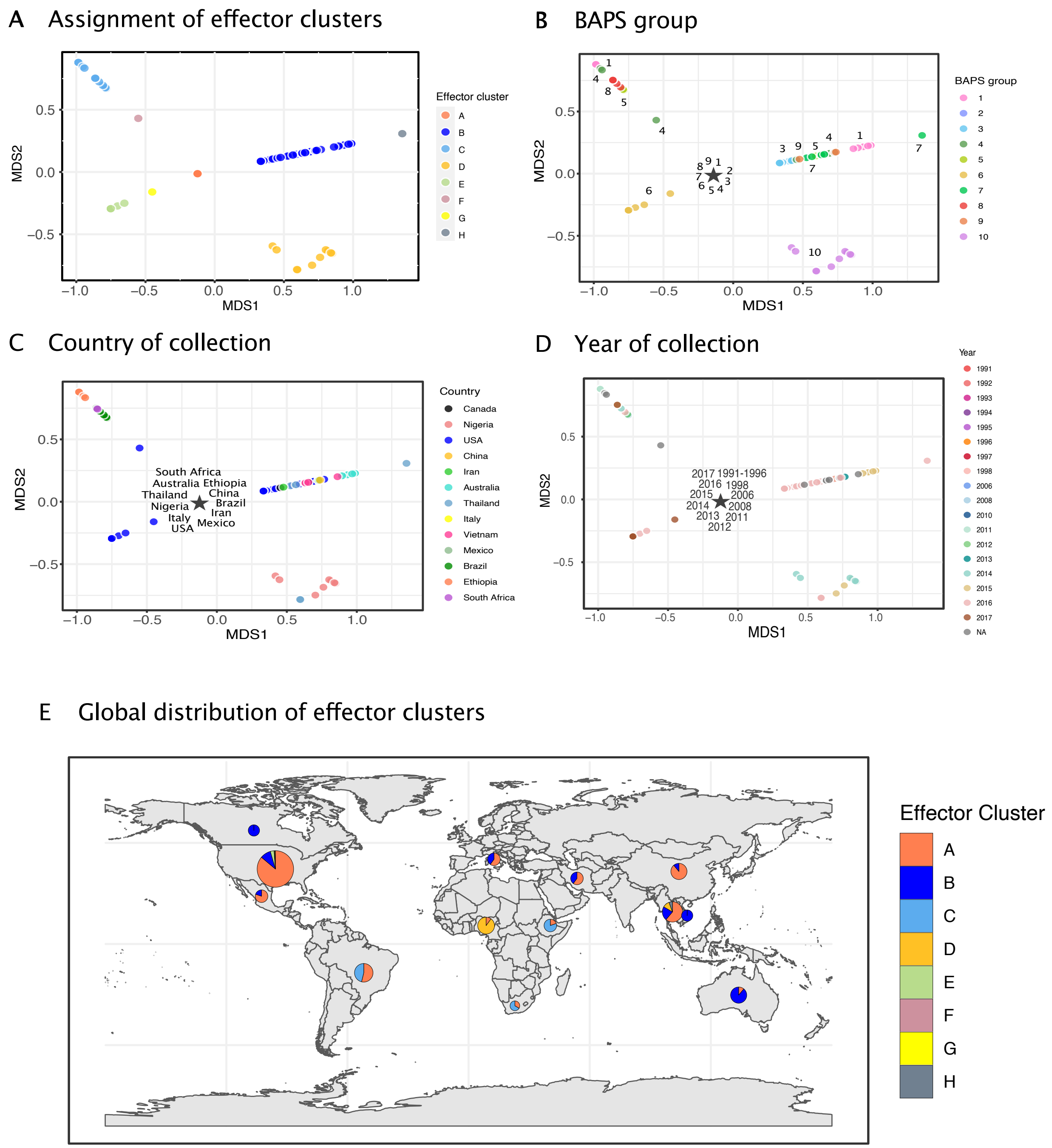

S6 Figure. Clustering of 270 *Xanthomonas perforans* effector profiles by non-metric multidimensional scaling and distribution of resulting clusters among geographic regions. Analysis did not include TAL effectors. (A) The most frequently observed group of effector profiles form cluster A. This cluster of 188 strains is represented as a star in plots B-C, as it is represented in most BAPS core gene clusters (B), most of the sampled tomato production regions (C), and in collections from 1991 to 2017 (D). Clusters were largely defined by low frequency effectors (S7 Figure). (E) Distribution of strains by effector clusters among sampled countries.
