# Supplementary material for: Diversification of an emerging bacterial plant pathogen; insights into the global spread of Xanthomonas euvesicatoria pv. perforans
Source: PLoS Pathog. 2025 Apr 9;21(4):e1013036. doi: 10.1371/journal.ppat.1013036 (PMC12047805; doi:10.1371/journal.ppat.1013036)
Supplement: S7 Fig — Analysis did not include TAL effectors. Type III effectors are in columns and Xep strains in rows. Effector status is shown by allele type: absence is indicated by allele type 0 (white), while the most frequent allele observed when the effector is present is allele type 1 (purple), second most frequent is allele type 2 (blue), and so on. Putative pseudogenized effectors are shown as allele 13 (gray). The order of columns was determined by hierarchical clustering analysis, placing similarly distributed effectors adjacent to each other. Order of rows is based on NMDS clustering analysis of effector profiles (see S6 Fig). (PDF) [file ppat.1013036.s007.pdf]

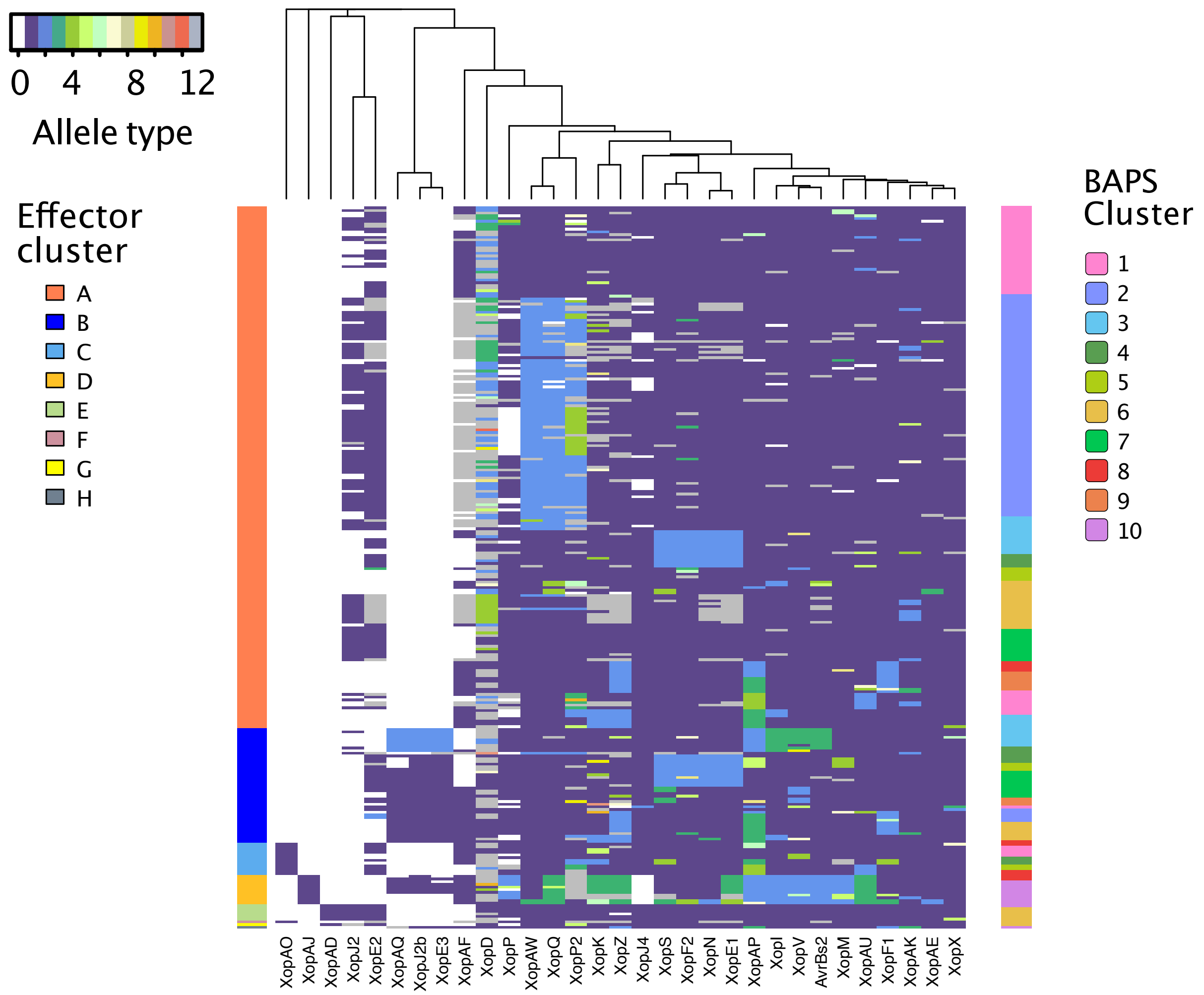

S7 Figure. Variation in Type III secreted effector profiles in 270 *Xanthomonas euvesicatoria* pv. *perforans* strains ordered according to NMDS of effector profiles. Analysis did not include TAL effectors. Type III secreted effectors are in columns and *Xep* strains in rows. Effector status is shown by allele type: absence is indicated by allele type 0 (white), while the most frequent allele observed when the effector is present is allele type 1 (purple), second most frequent is allele type 2 (blue), and so on. Putative pseudogenized effectors are shown as allele 13 (gray). The order of columns was determined by hierarchical clustering analysis, placing similarly distributed effectors adjacent to each other. Order of rows is based on NMDS clustering analysis of effector profiles (see S6 Figure, part A).
